# Ammonia and salinity tolerance of *Penaeus monodon* across eight breeding families

**DOI:** 10.1186/s40064-016-1878-1

**Published:** 2016-02-25

**Authors:** Jinsong Chen, Falin Zhou, Jianhua Huang, Zhenhua Ma, Shigui Jiang, Lihua Qiu, Jian G. Qin

**Affiliations:** South China Sea Fisheries Research Institute, Chinese Academy of Fishery Sciences, Guangzhou, 510300 China; Key Laboratory of South China Sea Fishery Resources Exploitation and Utilization, Ministry of Agriculture, Guangzhou, 510300 China; School of Biological Sciences, Flinders University, GPO Box 2100, Adelaide, SA 5001 Australia

**Keywords:** Ammonia nitrogen, Salinity, Stress, *Penaeus monodon*

## Abstract

Ammonia nitrogen and salinity tolerance of *Penaeus monodon* from eight selected breeding families were evaluated at the concentration of 67.65 mg L^−1^ ammonia-N and reducing salinity from 15 to 0 ‰. The final survival of family A (88.67 ± 9.81 %) was highest, and the final survival of family B was lowest (24.33 ± 14.01 %) after the ammonia tolerance test. Upon completing the sudden drop salinity test from 15 to 0 ‰, the highest survival was observed in family B (98.00 ± 1.73 %), and the lowest survival was found in family H (18.00 ± 1.73 %). Family A showed the strongest ability to tolerate ammonia stress, and family B showed the strongest tolerance to low salinity. This study suggests that the tolerance of salinity and ammonia nitrogen varied between breeding families. Results from the present study provide useful information towards selective breeding in shrimp in aquaculture for environmental tolerance.

## Background

During the whole life history, aquatic organisms inevitably experience environmental variation and stress such as temperature, pH, light, oxygen, and salinity. Ammonia is toxic to aquatic animals and can cause damage in gills, skin and blood circulation (Wang et al. 2006). Ammonia nitrogen is the major pollutant in the aquaculture environment, and is one of the most important water quality parameters to monitor (Yue et al. 2010). Therefore, accumulation of these toxicants and their toxic effects are the primary concern in the intensive system for shrimp culture (Chen et al. 1990). In a closed aquaculture system, ammonia is one of the main concerns as it may accumulate over time, primarily from the decomposition of organics and from the brachial excretion of the culture animals. High concentrations of ammonia may result in retardation of shrimp growth, and in extreme cases can cause shrimp mortality (Ostrensky and Wasielesky 1995; Alcaraz et al. 1999) demonstrate that *Penaeus setiferus* postlarvae are sensitive to ammonia and nitrite, and mass mortality may occur within 24 h when shrimp exposing to 268.06 mg L^−1^ nitrite-N. Wang et al. (2006) found that the activity of antioxidant enzymes in *Penaeus vannamei* decreased with the increasing of ammonia-N concentration. Depressed activity of antioxidant enzymes can cause oxidative damage, and affecting the immune system in shrimp (Wang et al. 2006). Huang et al. (2006) found that with the increase of ammonia nitrogen stressing concentration and time, the nonspecific immune defense system of shrimp was destroyed.

Salinity is another important abiotic factor in aquaculture (Ye et al. 2009), affecting physiological activities in aquatic organisms (Romano and Zeng 2012). In aquatic organisms, osmoregulatory adaptive mechanisms have been developed to survive in different salinity conditions (Nikapitiya et al. 2014). Previous studies have demonstrated that the salinity tolerance of *Penaeus monodon* (Fabricius 1798) ranged from 1 to 57 ‰, and the most suitable salinity range for growth was from 10 to 35 ‰ (Ye et al. 2009; Wang and Chen 2006). In the outdoor rearing ponds, the fluctuation of salinity is a primary reason causing failure of shrimp production. This is because salinity changes over a particular range can affect the immune system of shrimp and make them highly vulnerable to pathogens (Nikapitiya et al. 2014). Drastic salinity changes may also affect the feed intake, metabolism, and higher-energy utilization for osmoregulation resulting in poor growth (Joseph and Philip 2007). Therefore, pedigree selecting of resistance to drastic salinity change is desired in shrimp aquaculture.

Tiger shrimp *P. monodon* is one of the most important species of *Penaeus* currently being cultured commercially in many countries, especially in Southeast Asia. Currently, ammonia nitrogen and salinity are the major abiotic factors affecting the growth and survival of *P. monodon* in commercial production. In order to increase production efficiency, select the trait of more tolerance to high ammonia and low salinity is desired in *P. monodon* farming. To evaluate the resistance of ammonia nitrogen stress and salinity stress, eight *P.monodon* families were selected to compare in this study. Results from the present study will provide valuable information to guide selective breeding for *P.monodon* in aquaculture.

## Methods

### Experimental animals and rearing condition

Post larvae of *P. monodon*, averaging 0.94 ± 0.42 g in body mass, 4.17 ± 0.71 cm in body length were cultured in outdoor tanks at the Shenzhen Base of South China Sea Fisheries Research Institute, People’s Republic of China. Continuous aeration was provided into the rearing tanks, and shrimps were fed on a commercial shrimp diet (Shuang-hu Feed Limited, Guangdong, China). The dietary compositions are as follows: 41.0 % crude protein, 4.0 % crude lipid, 16.0 % ash, 5.0 % crude fibre and 12.0 % moisture. The salinity of the sand-filtered seawater was 30 ‰, and the water temperature was maintained at 29 ± 0.5 °C. Eight families of *P. monodon* from two strains (South China Sea No. 1 strain and Africa strain) were selected as experimental materials (Table 1). The experiments were conducted in accordance with the guidelines and approval of the Ethics Committee of South China Sea Fisheries Research Institute, Chinese Academy of Fishery Sciences (2012AA10A409).

**Table 1:** Families information of eight selected *P. monodon* families

Family ID	Parents ID (♀ × ♂)	Strains (♀ × ♂)
A	C 663 × F 033	S × S
B	K 090 × E 613	S × S
C	J 312 × E 488	S × A
D	J 084 × E 638	S × A
E	E 134 × I 008	A × S
F	E 021 × H 579	A × S
G	E 587 × E 688	A × A
H	E 942 × E 840	A × A

Different characters and numbers in the Parents ID were the individual shrimp

*S* South China Sea No. 1 strain was originally come from South China Sea, and was bred and selected based on growth traits (animals used in this study was the fourth generation), *A* African strains were imported from Mozambique, Africa, and was artificially bred in our research station (animals used in this study was the fourth generation)

### Experimental design and set-up

#### Ammonia nitrogen stress

To determine the LC_50_ (medial lethal concentration), a total of six ammonia-N concentrations, including 0, 20, 40, 60, 80, and 100 mg L^−1^ were tested in the preliminary trial. All the experiment condition and operating protocol were same as mentioned above. Shrimps with same size were tested in each concentration of ammonia-N in triplicates. During the preliminary trial, mortality was recorded every 1 h. Upon on completing the preliminary trial, mortality of shrimp was calculated at 24, 36, 48, 72, and 96 h in each concentration of ammonia-N. Then use the concentration of ammonia-N as X-axis and mortality as Y-axis to establish the regression equation of straight line: y^ = a + b x, and calculate the concentration of ammonia-N when the mortality was 50 % (Li et al. 2012; Norberg-King 1993).

After the 96 h preliminary experiment, mortality of shrimp was recorded, and an LC_50_ concentration of 67.65 mg L^−1^ ammonia-N was determined by linear interpolation. The ammonia solutions were prepared by adding ammonium chloride (NH_4_Cl) to freshwater until the desired concentration was attained. During the experimental period, the water temperature was 29 ± 0.5 °C, the pH was 7.8, and the salinity was 30 ‰. Shrimps with similar size from eight families were stocked into the experimental tanks containing 20 L of seawater (30 shrimps per tank). For each selected family, three replicates were tested during this study, and a total of 720 shrimps were used in this study. Water in the experimental tanks was replaced at 50 % volume every 12 h. The survival of test shrimps in each tank was recorded every 2 h. During the experiment, no food was supplied to the tanks, and the dead shrimps were removed from the tank upon detected.

#### Salinity stress

The experimental condition was as same as above. To achieve the desired salinity level, the seawater and fresh water (tap water filtered by sand filter) were mixed to adjust the level of salinity. The salinity was measured by high-precision salinometer (ATAGO, Guangdong, China). Our preliminary study indicated that reducing salinity from 30 to 15 ‰ had no effects on the survival of testing shrimps from eight selected families. Therefore, testing shrimps were directly exposure to 15 ‰ when experiment was started. This test lasted 72 h, and the salinity was dropped every 24 h. The salinity was reduced from 15 to 1 ‰ within 24 h, and further reduced from 1 to 0 ‰ in 24 h. Upon the salinity reached to 0 ‰, the survival of test shrimps in each tank was determined every 2 h for the following 24 h. No feeding during the test, and the dead shrimps were collected every 2 h.

### Data analysis

Data are presented as the mean ± SD in this study. Statistical comparisons of experimental data were performed by one-way analysis of variance (ANOVA) using the software SPSS 18.0 and Duncan Multiple Range test was used to identify significant differences among the families. The level of statistical significance was set at *P* < 0.05.

## Results and discussion

Family selection is a traditional breeding method that the whole family is considered as a selection unit, and according to the size of the average family traits to determine whether individuals are select for or against. Family selection is applicable to phenotypic trait of low heritability, such as fecundity, survival rate, and resistance. He et al. (2008) evaluate the ammonia nitrogen resistance of larvae of *Panaeus. chinensis* base on the average LD_50_ of 24, 48 and 72 h. Eight families of *P. chinensis* were selected for their ability to tolerate ammonia nitrogen. Sun et al. (2011) use the 48 h LC_50_ (43 mg L^−1^) to evaluate 13 juvenile (3.46 ± 0.10 g) *P. monodon* families, and find that the ammonia nitrogen tolerance of different family is significantly different (*P* < 0.05). The LC_50_ of nitrite-N in the present experiment was 67.65 mg L^−1^. In the present study, with the increasing of experimental duration, the survival of each family significantly decreased (*P* < 0.05, Table 2). After ammonia nitrogen stress for 96 h, the final survival of tested families ranged from 23.00 ± 10.00 % to 88.67 ± 9.81 %. The final survival of family A (88.67 ± 9.81 %) was highest, and the final survival of family H was lowest, only was 23.00 ± 10.00 % survived during the test (Table 2).This may suggest that *P. monodon* families may be a strain that has the potential of high ammonia nitrogen tolerance.

**Table 2:** Cumulative survival (% mean ± SD) of eight selected families at different exposure time during the ammonia nitrogen stress

Family	Time (h)			
	24	48	72	96
A	100.00 ± 0.00^c^	91.33 ± 7.51^d^	91.33 ± 7.51^d^	88.67 ± 9.81^d^
B	91.00 ± 1.73^c^	64.33 ± 10.26^abc^	42.33 ± 21.36^ab^	24.33 ± 14.01^ab^
C	97.00 ± 0.00^c^	74.33 ± 19.63^bcd^	66.67 ± 20.31^bcd^	55.33 ± 21.36^c^
D	87.67 ± 5.03^bc^	68.67 ± 5.13^abc^	51.00 ± 8.54^abc^	32.33 ± 9.24^abc^
E	86.67 ± 12.34^bc^	62.33 ± 12.86^ab^	52.33 ± 15.28^abc^	32.33 ± 12.86^abc^
F	71.33 ± 12.50^a^	53.33 ± 15.28^bc^	44.33 ± 20.13^ab^	33.33 ± 20.31^abc^
G	100.00 ± 0.00^c^	85.67 ± 9.81^cd^	72.33 ± 5.03^cd^	51.33 ± 12.50^bc^
H	74.33 ± 10.26^bc^	50.33 ± 5.77^a^	37.67 ± 5.03^a^	23.00 ± 10.00^a^

Values (expressed as mean ± SD, n = 3) with different letters in the same column are significantly different from each other (*P* < 0.05)

Salinity influence on the respiratory metabolism of *Penaeus* is usually thought to be an energy consumption adjustment caused by an osmotic pressure difference between the environment and body fluid (Ye et al. 2009). Most penaeid shrimps are known to be euryhaline species growing in a wide range of salinities. *P. monodon* exhibits hyper-osmotic regulation at low salinity levels, and exhibits hypo-osmotic regulation at high salinity levels (Cheng and Liao 1986). In the present study, lower salinity significantly affected the survival of shrimps in all the tested families (Table 3), and lower salinity tends to be more stressful for *P. monodon* as observed. In this study, no shrimp died at the salinity of 15 ‰. After the salinity was reduced to 1 ‰, the survival of selected families was in the range of 71.00 ± 3.46 % to 99.00 ± 1.73 %, and showed significant difference among families (*P* < 0.05). The survival of family B (99.00 % ± 1.73 %) was highest, and the survival of family A was lowest in which only 71.00 % ± 3.46 % was survived. After the salinity was reduced to 0 ‰, the survival of selected families ranged from 18.00 ± 1.73 % to 98.00 ± 1.73 %. Upon completing the experiment, the highest survival was observed in family B (98.00 ± 1.73 %), and the lowest survival was found in family H (18.00 ± 1.73 %, Table 3). The results of the current experiments indicated that salinity can have an immediate and significant effect on survival of *P. monodon*. All families survived at the salinity of 15 ‰, and the low survival after the salinity dropped to 0 ‰ found in this study, which was similar to the result obtained by Ye et al. (2009).

**Table 3:** Cumulative survival (%) of eight selected families at different salinity (mean ± SD)

Family	Salinity (‰)		
	15	1	0
A	100.00 ± 0.00	71.00 ± 3.46^a^	63.33 ± 5.77^c^
B	100.00 ± 0.00	99.00 ± 1.73^d^	98.00 ± 1.73^d^
C	100.00 ± 0.00	89.00 ± 1.73^bc^	56.67 ± 9.07^bc^
D	100.00 ± 0.00	87.67 ± 4.04^bc^	26.67 ± 3.51^a^
E	100.00 ± 0.00	91.00 ± 8.54 ^cd^	47.67 ± 6.81^b^
F	100.00 ± 0.00	88.67 ± 5.13^bc^	44.00 ± 10.39^b^
G	100.00 ± 0.00	95.33 ± 4.04^cd^	87.67 ± 10.79^d^
H	100.00 ± 0.00	80.00 ± 8.89^ab^	18.00 ± 1.73^a^

Values (expressed as mean ± SD, n = 3) with different letters in the same column are significantly different from each other (*P* < 0.05)

## Conclusions

Eight selected *P.monodon* families were compared to evaluate the resistance of ammonia nitrogen stress and salinity stress in this study. Significant difference of final survival of the ammonia nitrogen stress and salinity stress among families was found in this study. In the present study, the survival of eight families was significantly different after the salinity dropped to 0 ‰. This result has significant implications for *P. monodon* aquaculture, as it can be utilized in family selection, and salinity maintenance to maximize commercial productivity. Family A was the strongest family in the ammonia nitrogen tolerance test, and family B was the strongest family in the low salinity tolerance test. All the parents of family A and family B came from “South China Sea strains”, it proved that the “South China Sea strains” have the higher resistance to adverse environmental elements and it is the strong breed of *P.monodon*. Future selective breeding should be toward screening and purifying this type family.
